# Interstitial High-Dose-Rate Brachytherapy of Liver Metastases in Oligometastatic Patients

**DOI:** 10.3390/cancers13246250

**Published:** 2021-12-13

**Authors:** Franziska Walter, Maya Rottler, Lukas Nierer, Guillaume Landry, Justus Well, Paul Rogowski, Konrad Mohnike, Max Seidensticker, Jens Ricke, Claus Belka, Stefanie Corradini

**Affiliations:** 1 Department of Radiation Oncology, University Hospital, Ludwig Maximilian University, 81377 Munich, Germany; Franziska.walter@med.uni-muenchen.de (F.W.); maya.rottler@med.uni-muenchen.de (M.R.); Lukas.nierer@med.uni-muenchen.de (L.N.); guillaume.landry@med.uni-muenchen.de (G.L.); Justus.well@med.uni-muenchen.de (J.W.); paul.rogowski@med.uni-muenchen.de (P.R.); claus.belka@med.uni-muenchen.de (C.B.); 2 Diagnostisch Therapeutisches Zentrum (DTZ), 10243 Berlin, Germany; konrad.mohnike@berlin-dtz.de; 3 Department of Radiology, University Hospital, Ludwig Maximilian University, 81377 Munich, Germany; Max.seidensticker@med.uni-muenchen.de (M.S.); jens.ricke@med.uni-muenchen.de (J.R.)

**Keywords:** liver, metastases, brachytherapy, radiotherapy, local control, survival, outcome

## Abstract

**Simple Summary:**

Local ablative treatments have emerged as a promising treatment strategy for patients with oligometastatic disease. Interstitial brachytherapy (iBT) is one of the locally ablative treatment options for unresectable liver metastases in oligometastatic disease. We report the feasibility and oncologic outcome of 141 iBT treatments of 244 oligometastatic liver metastases performed in patients with limited tumor burdens in a high-volume center. iBT was feasible, safe and effective in the treatment of oligometastatic liver metastases with good local control rates and low toxicity. Histology and total tumor volume had an impact on local control rates.

**Abstract:**

Local ablative treatments have emerged as a promising treatment strategy for patients with oligometastatic disease. Among others, interstitial brachytherapy (iBT) is an upcoming treatment option for unresectable liver metastases. We report the feasibility and oncologic outcome of iBT of oligometastatic liver metastases performed in patients with limited tumor burdens in a high-volume center. Patients undergoing iBT between August 2017and March 2019 were included. A retrospective analysis of patient outcomes and treatment complications was performed. Patients treated for metastatic colorectal carcinoma (CRC) were compared to other histologies. A total of 141 iBT procedures were performed in 106 patients (male:52; female:54) and 244 liver metastases. Overall, 51% (54/106) of patients had a diagnosis of metastatic CRC. The median follow-up was 9 months, and overall survival (OS) was 92.3% at 6 months and 76.3% at 12 months. Local-relapse-free survival (LRFS) was 88.4% at 6 months and 71.5% at 12 months, with a significant difference between patients with CRC (84.1% and 50.6%) versus other histologies (92.4% and 92.4%, *p* < 0.001). A sub-group analysis showed a significant advantage in patients with CRC receiving a minimal dose (D100) of 20 Gy to the planning target volume. Treatments of smaller total liver-tumor volumes (<18 ccm) resulted in better LRFS rates. iBT is a safe and effective treatment approach for oligometastatic liver disease. A higher treatment dose is needed for patients with CRC. Moreover, lower metastatic burdens may be favorable for LRFS. Prospective studies are needed to assess the role of iBT in the oligometastatic setting as an alternative to other local ablative treatment approaches in patients with liver metastases.

## 1. Introduction

Patients with solid tumors who develop distant metastases with a low metastatic burden represent a unique clinical scenario that requires an adaption and optimization of traditional treatment strategies. The term “oligometastatic disease” has become well-established in recent years. While the exact definition of oligometastatic disease remains controversial, with several recommendations being published [1,2], there is general agreement that more aggressive local treatments are desirable in patients with a low metastatic burden. Oncologic benefits of this strategy have been found for several cancer entities. In patients with metastatic prostate cancer, metastasis-directed therapies can prolong androgen-deprivation-therapy-free survival [3]. Similarly, a recent randomized study investigated the use of radiation-based local ablative treatments using stereotactic ablative radiotherapy (SABR) for the treatment of oligometastatic disease of various histologies. The study found an increase in overall survival (OS) and progression-free survival (PFS) in patients with locally controlled primary tumors and one to five distant metastases [4].

Moreover, the development of highly potent systemic therapies has contributed to a paradigm shift from the exclusively palliative status of a metastasized disease to a potentially curable condition in an increasing number of patients with solid tumors. This has renewed the interest in locally ablative treatment options for patients with limited metastatic burdens in combination with systemic treatments. A randomized study showed a PFS benefit of SABR to all macroscopic tumor sites, in addition to maintenance chemotherapy in patients with limited metastatic non-small-cell lung cancer (NSCLC) over maintenance chemotherapy alone [5]. A second study even showed prolonged OS [6].

The concept of metastasis-directed therapy has especially been established in patients with colorectal cancer (CRC) pre-treated with effective systemic therapy [7]. The resection of lung and liver metastases is the gold standard in patients eligible for radical surgery [8,9]. However, in cases of unresectable metastases, a variety of other local ablative treatments, the so-called “toolbox of local ablative treatments”, can be applied [10]. In a phase II trial, a prolonged OS was reported for patients undergoing thermal ablation of unresectable liver metastases versus systemic treatment alone [11].

Regarding radiation-based techniques for the treatment of liver metastases, two established modalities are currently available: either external beam radiation using SABR, or interventional radiotherapy using minimally-invasive interstitial high-dose-rate brachytherapy (iBT) [12]. SABR is a widely available technique that is frequently employed in the treatment of a variety of anatomic regions, such as brain metastases [13], lung metastases [14], liver metastases [15], and bone metastases [16]. In contrast, interstitial HDR brachytherapy of the liver is generally underutilized, although brachytherapy is a well-established radiation technique. iBT is an excellent tool to deliver very high radiation doses directly to liver lesions with little morbidity. However, the current literature on liver brachytherapy is sparse due to the limited applications of the technique, and usually consists of single-center experiences with a limited number of patients. We present our experience as a high-volume center with a total of 244 liver metastases treated in patients with oligometastatic disease from various primary tumors. We evaluated the clinical outcomes of these patients, as well as the feasibility and tolerability of iBT for liver metastases. Specifically, we evaluated patients undergoing liver iBT for metastatic CRC versus other histologies and explored other characteristics that might influence local control rates.

## 2. Materials and Methods

The study was performed in accordance with the Declaration of Helsinki in its latest version and was approved by the Ethics Committee, LMU Germany (LMU-18-511).

### 2.1. Patients

A retrospective analysis was performed, including patients treated with iBT in liver lesions between August 2017 and March 2019. All patients were discussed by an interdisciplinary tumor board before treatment and were deemed amenable for local ablative treatment due to low metastatic burdens. Only patients receiving iBT of up to 5 lesions in one session were included in this retrospective analysis. Patients undergoing iBT of the liver for either hepatocellular carcinoma or cholangiocellular carcinoma were excluded from the analysis. Patient and treatment characteristics were extracted from clinical reports and by review of the treatment plans.

### 2.2. Brachytherapy

An experienced interventional radiologist performed the placement of the brachytherapy catheters under CT fluoroscopy. Patients were positioned with their arms above their heads. The metastases were punctured using an 18-gauge needle. A stiff guidewire was used to change to an angiography sheath with a 6F diameter (Radifocus, Terumo, Japan). Finally, the brachytherapy catheter (Primed Halberstadt Medizintechnik, Halberstadt, Germany) was placed in the sheath. The number of inserted catheters was dependent on tumor number and size. With the catheters in place, a multislice CT was acquired with a slice thickness of 2 mm. If applicable, intravenous (i.v.) contrast was administered. For treatment planning, the CT dataset was transferred to the treatment-planning software Oncentra Brachy (Elekta AB, Stockholm, Sweden) version 4.5.2.

Total liver volume and directly adjacent organs at risk (OAR) such as the stomach, duodenum, bowel, heart or kidney were contoured. A radiation oncologist defined the clinical target volume (CTV) using additional pre-treatment diagnostic imaging (CT with i.v. contrast or liver-specific MRI with hepatocyte-specific contrast). No addition of a setup margin is necessary in iBT; therefore, the CTV was identical to the planning target volume (PTV). The dose was prescribed to cover 100% of the volume of the PTV (D100). Usually, a dose of 20 Gy was prescribed to the D100, with the exception of patients with metastatic CRC. In these cases, a dose of 25 Gy was prescribed, according to the dose-finding study of Ricke et al. [17]. However, sparing of OARs with fulfilment of the corresponding dose constraints always had priority over full dose coverage. Compared to other entities, dose distributions in liver brachytherapy plans are usually inhomogeneous with very high central doses.

**Figure 1:**
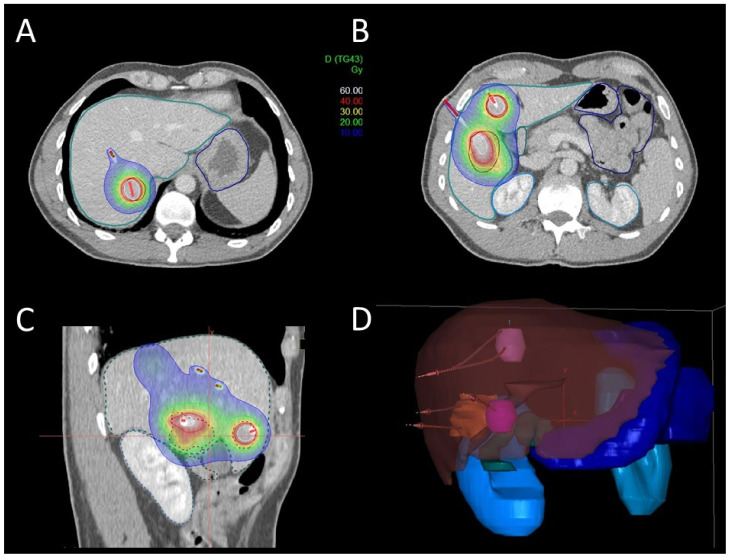
Patient example of interstitial brachytherapy in multiple lesions. (**A**,**B**) Axial views of multiple liver lesions in different liver segments; (**C**) sagittal view of multiple liver lesions; (**D**) reconstruction of liver volume with multiple lesions and multiple catheters in place.

Catheter reconstruction was performed by a medical physicist using the hyperdense catheter tip as a reference point. For dose optimization, dwell points were placed with 2 mm distance along the length of the catheter. Dose plan optimization was performed manually using the Oncentra Brachy v4.5.2. planning system (graphical and manual dwell-time optimization). It utilizes dose calculation according to the AAPM TG-43U1 formalism. Organs-at-risk dose constraints were given as follows: bowel/colon and stomach D1 ccm: <12 Gy, D0.1 ccm: <15 Gy; esophagus D1 ccm: <12 Gy, D0.01 ccm: <15 Gy; spinal cord D0.01 ccm: <10 Gy; skin D0.01 ccm: <10 Gy. One-third of the uninvolved liver (liver-PTV) was irradiated with less than 5 Gy. A clinical example is shown in Figure 1. The volume of all target lesions was added for each patient in all treatment sessions (sum of all PTV volumes). Mean and median treated total volume was calculated.

### 2.3. Follow-Up and Treatment Complications

The first follow-up was performed within 90 days of the end of treatment, and included an MRI or CT of the liver. The subsequent follow-up was performed every three months for the first two years after BT, and every six months afterward. Complications following treatment within 6 months after iBT were identified retrospectively.

### 2.4. Statistics

Overall survival (OS) was calculated from the day of the iBT treatment to death or last follow-up (FU). Local-relapse-free survival (LRFS) was defined as time from the day of iBT to a radiologically confirmed progression within a treated lesion or last FU. PFS was calculated from the day of iBT to a local or distant progression (whichever occurred first) or last FU. OS, LRFS and PFS were calculated per patient (taking into account that some patients underwent multiple iBT treatment sessions) using the Kaplan–Meier method. OS, LRFS and PFS were compared for patients with CRC vs. other histologies (“mixed histology”) using the logrank test. A sub-group analysis was performed in patients with CRC, comparing patients who received at least a dose of D100 ≥ 20 Gy in all PTVs in every iBT session to patients who received a lower dose in at least one PTV. Finally, LRFS was calculated for treated volume per patient above and below the median of the treated volume of the entire cohort using the Kaplan–Meier method.

## 3. Results

### 3.1. Patients

Between August 2017 and March 2019, we performed 282 iBT treatments of 545 liver lesions. Of these, 29 iBT treatments were applied in patients with CCC, 86 in patients with HCC and 167 in patients with liver metastases. A total of 141 iBT treatments were performed in 106 patients (m:52; f:54) who met the inclusion criteria with a maximum of 5 lesions treated per iBT session and were included in the current analysis.

A detailed overview of patient and treatment characteristics is presented in Table 1. Liver metastases from a variety of primary tumors were included. The most common histology was CRC liver metastasis in 51% (54/106) of patients treated in a total of 76 treatment sessions (rectosigmoid cancer *n* = 39, colon cancer *n* = 15). Moreover, 42% (45/106) of patients also had a metastatic burden outside of the liver at the time of brachytherapy.

Concerning additional treatment modalities, 56 patients (52%) underwent further local treatments such as surgery, radiofrequency ablation (RFA), transcatheter arterial chemoembolization (TACE), microwave ablation (MWA) or selective internal radiation therapy (SIRT) following iBT. Additionally, 78 patients received systemic therapy after undergoing iBT of the liver. Unfortunately, for nine patients, no detailed information was available confirming if subsequent systemic treatment was administered.

### 3.2. Brachytherapy

A total of 141 treatment sessions were evaluated. In 72 cases, 1 lesion was treated; in 44 cases, 2 lesions; in 17 cases, 3 lesions; in 7 cases, 4 lesions and, in one case, 5 lesions were treated within the same treatment session. Overall, a total of 244 lesions were treated. The mean metastasis diameter was 3.2 ± 1.9 cm, and the mean metastasis volume was 23.3 ± 58.1 ccm. The prescribed dose to the PTV was either 20 Gy, or 25 Gy in cases of CRC histology. The mean applied dose D100 was 19.3 ± 4.8 Gy, D98: 23.0 ± 5.7, D95: 25.1 ± 6.4 Gy and D90: 27.9 ± 7.3 Gy. The mean treated volume per patient was 53.0 ± 92.5 ccm and the median volume was 18.4 ccm (range 0.4–615.1 ccm).

### 3.3. Treatment Complications

Two patients (1.8%) developed an abscess of the liver, one of whom had undergone pancreaticoduodenectomy with a biliodigestive anastomosis, which is a known risk factor. Both liver abscesses occurred with a delay of several months after the iBT treatment. One patient (0.9%) developed segmental cholestasis within the 4 weeks after iBT, which did not require an intervention, and one patient (0.9%) had a major bleeding event after removal of the BT catheters. This patient developed a hematoma that required embolization; however, no blood transfusion was necessary.

### 3.4. Follow-Up

**Figure 2:**
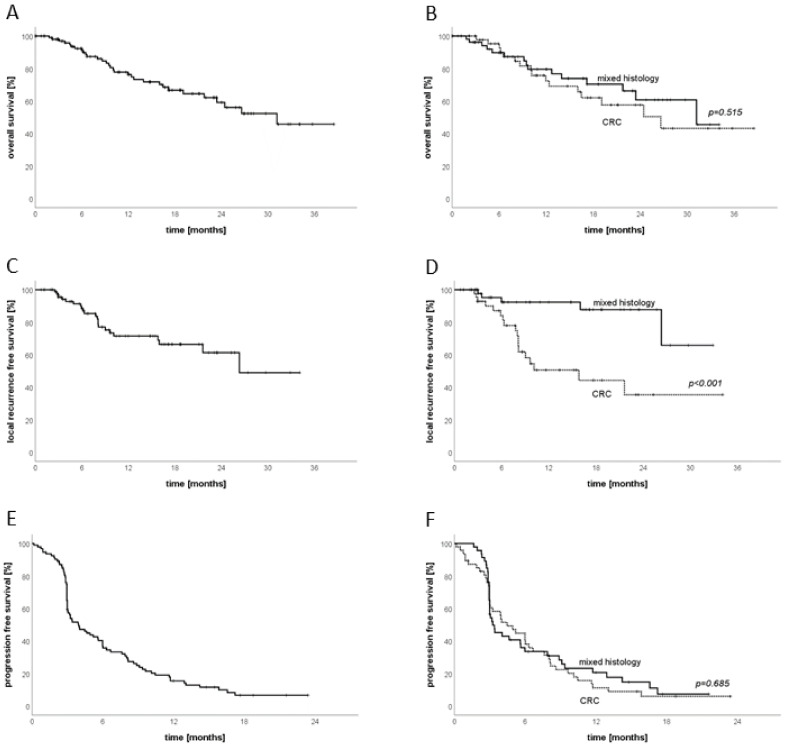
Oncologic outcome of interstitial brachytherapy. (**A**) Overall survival; (**B**) overall survival CRC vs. mixed histology; (**C**) local-recurrence-free survival; (**D**) local-recurrence-free survival CRC vs. mixed histology; (**E**) progression-free survival; (**F**) progression-free survival CRC vs. mixed histology.

The median follow-up was 9 months (range: 3–39 months). The OS for the entire cohort after 6 months and 12 months was 92.3% and 76.3%, respectively. Analyzed by histology, the OS for patients with CRC was 95.2% and 72.4%, respectively, while in patients with mixed histology, it was 89.7% and 79.6%, with no significant difference between the two groups (*p* = 0.515). The LRFS of the entire cohort reached 88.4% after 6 months and 71.5% after one year, with a significant difference between patients with CRC (84.1% and 50.6%) and patients with mixed histology (92.4% and 92.4%, *p* < 0.001). Concerning PFS, the entire cohort had a PFS of 36.8% and 15.5% after 6 and 12 months, respectively. The difference between histologies was not significant, with CRC reaching 40.3% and 11.2%, and mixed histology 33.5% and 20.6%, respectively; *p* = 0.685 (Figure 2).

### 3.5. Sub-Group Analysis CRC

**Figure 3:**
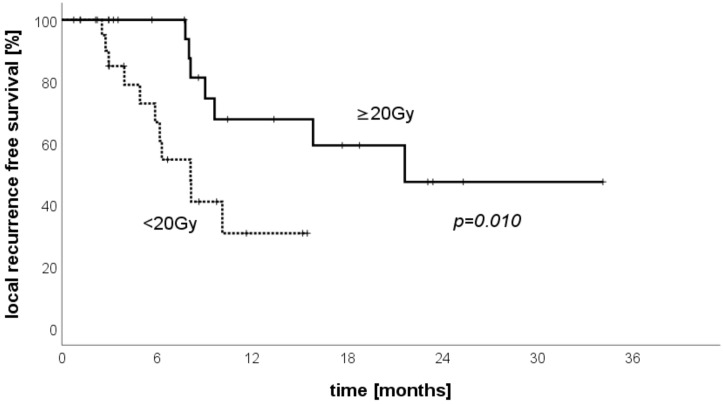
Local-recurrence-free survival for histology of colorectal carcinoma (CRS) stratified by dose.

We performed a dedicated analysis of LRFS in the sub-group of patients treated for metastases of CRC stratified by dose applied in the D100. Those patients who received a D100 of ≥20 Gy in all treated lesions and in all applied fractions had a significantly better LRFS than patients with a lesser dose applied (Figure 3). The LRFS after 6 months was 100% vs. 66.8%, and the one-year LRFS was 47.7% versus 30.7% in this sub-group (*p* = 0.010).

### 3.6. LRFS Stratified by Treated Volume per Patient

**Figure 4:**
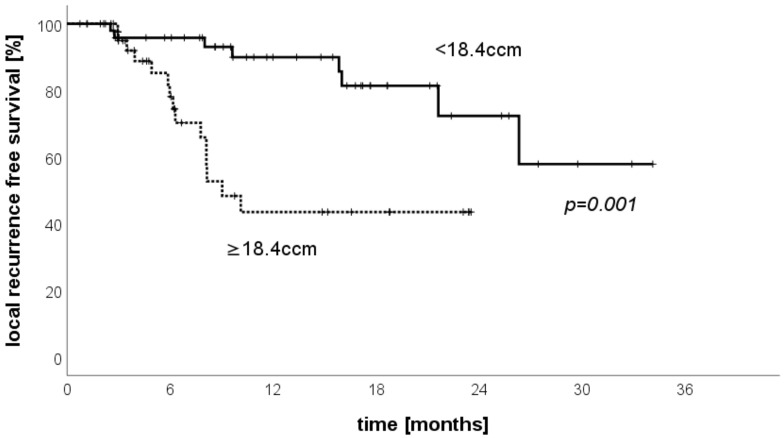
Local-recurrence-free survival stratified by total treated volume.

We also performed an analysis concerning the LRFS stratified by the total treated volume. The median treated volume per patient was 18.4 ccm. Therefore, the group of patients with treated volumes smaller than 18.4 ccm was compared to the group of patients with a volume of ≥18.4 ccm. The evaluation showed a statistically significantly better LRFS for patients with smaller tumor burdens (Figure 4, *p* = 0.001).

## 4. Discussion

Our results show that interventional radiotherapy using iBT is an effective treatment option for liver metastases in patients with low metastatic burdens from a variety of primary tumors. In the past, concerns about liver toxicity hampered the application of radiation treatments for the management of liver malignancies. However, since the introduction of SABR and the now-widespread accessibility of this technique, multiple studies have shown that radiotherapy is safe for the treatment of liver malignancies. In our study of 244 liver metastases, major treatment complications requiring an intervention (infection, bleeding, cholestasis) occurred in only four patients (3.7%) during 141 iBT treatments. In the literature, severe complications are rare, reported for less than 1.5% of all interventions, and include bleedings, infection and pain [18,19]. Regarding severe bleeding events, there is evidence of a strong correlation between major bleeding events and the secondary diagnosis of advanced liver cirrhosis (usually in patients with HCC), or the peri-interventional administration of low-molecular-weight heparin [20,21]. Liver abscesses (~2%) may occur more frequently in patients with a history of biliodigestive anastomosis, or following endoscopic papillectomy [20]. In summary, the complication rate is comparable to the present experience. Moreover, taking into consideration that more than 50% of our patients received other local treatments in addition to iBT, we can conclude that most patients had some degree of liver damage prior to our treatment. Patients referred to iBT for liver malignancies typically represent a negative selection for other, more-established local treatments, such as surgery. Therefore, we conclude that iBT performed at a specialized center is a safe treatment option for patients with liver metastases. To the best of our knowledge, this is the largest patient cohort reported from a single center so far. The available literature on interstitial liver brachytherapy is summarized in Table 2.

Most studies on iBT for liver malignancies focused on one histological tumor type at a time. In our cohort, we decided to include patients with metastases of different histologies. This allowed for a comparison of the outcome of all different histologies (“mixed histology”) vs. CRC as the most prevalent histology. The groups were comparable in size, with 52 vs. 54 patients. Based on the results of a prospective study that evaluated the dose-dependency of local tumor control in patients with CRC undergoing iBT for liver metastases [17], our clinical practice is to prescribe a dose of 25 Gy to metastases from CRC, while metastases of other solid tumors are treated with 20 Gy to the PTV. However, when we compared the clinical outcomes of patients with CRC versus other histologies, our data showed worse local control despite the higher prescribed doses, with an LRFS after 12 months of 50.6%. In comparison, Collettini et al. reported a local tumor control rate after 12 months of 88.3% after iBT on a cohort with 80 patients with metastatic CRC [23]. Our cohort consisted of patients not suitable for surgical resection or other local ablative techniques. We included a considerable number of patients with metastases in less-favorable localizations (close to OARs, central lesions). Therefore, a number of lesions could not be treated with the prescribed dose, but had a compromised dose to spare adjacent risk structures or to reduce overall liver exposure [36,37]. We conducted a sub-group analysis in patients with CRC that received a minimal dose of 20 Gy (D100) in all treated lesions in each individual fraction and compared it to patients with compromised dose coverage. This analysis clearly showed that patients with CRC treated with a sufficient radiation dose have significantly better local control rates than patients with de-escalated doses. This highlights the need to apply a sufficient radiation dose, especially in patients with metastatic CRC.

Kieszko et al. reported on 61 patients undergoing iBT for unresectable hepatic metastases of mixed histologies [31]. They reported OS rates after 6 months and 12 months of 96.7 and 79.6%, while the PFS rates were 78.1 and 53.8%, and the LRFS rates were 88.7 and 70.7%, respectively. The results for OS and LRFS are, overall, comparable to our results. However, in our cohort, the PFS was considerably worse, indicating that we included a higher percentage of patients with more-advanced disease. Within the group of mixed histologies, 10 out of 52 patients had metastatic pancreatic cancer. Considering the poor prognosis in terms of the PFS of these patients, this might have an influence on the overall PFS. In a report of 16 patients with pancreatic cancer undergoing iBT for liver metastases, either disease progression under chemotherapy or discontinuation of chemotherapy due to drug-related toxicity were the two main reasons for referral to local treatment. This study reports a local tumor control of 87% and a median PFS of only 3.4 months [34].

The term “oligometastatic disease” has not yet been finally defined. Several definitions have been proposed that take into account the absolute number of metastases and the number of involved organs. More recently, the question has been raised of whether the size of metastatic lesions should be included in the definition of oligometastatic disease [38]. We therefore evaluated the LRFS stratified by the total treated volume, and found a significant difference for patients treated for lesions less than 18 ccm, which was the mean total treated volume in our cohort, compared to patients treated for larger lesions. We found a significant advantage for patients undergoing iBT in the smaller total liver-tumor volumes. This seems to support the theory that not only the total count of metastases, but also the size and volume, should be incorporated into the decision to treat oligometastatic patients. The presented data, however, have some limitations, mostly inherent to the retrospective character of the evaluation. We did not perform post hoc verification of the oligometastatic status. In this cohort, oligometastatic disease was used as an umbrella term encompassing different clinical scenarios, such as initial oligometastatic disease, oligoprogression or oligorecurrence. However, all patients were discussed by interdisciplinary tumor boards prior to treatment, and the decision for the local ablative treatment was made for every patient in the context of their individual patient history. Another shortcoming is that detailed information on other treatment modalities—either systemic treatment or local ablative treatment—was not collected. Therefore, multivariate analysis of the available data was not possible. For this purpose, further prospective evaluations are needed.

## 5. Conclusions

iBT is a safe and effective treatment option for liver metastases that are not suitable for other local treatments. It provides good local control rates with few interventional treatment complications. A higher radiation dose is needed to effectively treat patients with oligometastatic CRC, and the overall volume of the treated metastases affects the local-control rate.

## Figures and Tables

**Table 1:** Patient and treatment characteristics.

Patient Characteristics	*n* = 106	Treatment Characteristics	*n* = 141
Age (years)	66 ± 12	Treated metastases	
Gender		1	72
Female	54	2	44
Male	52	3	17
Histology		4	7
Sarcoma	4	5	1
NET	7	Liver volume (mean, SD)	1466 ± 336 ccm
GIST	1	Liver exposure (mean, SD)	
Pituitary gland carcinoma	1	5 Gy	385 ± 310 ccm (26%)
Breast cancer	11	10 Gy	184 ± 169 ccm (13%)
Pancreatic cancer	10	15 Gy	110 ± 107 ccm (8%)
Gastric cancer	4	Catheters (number)	
Intestinal	1	1	35
Colon cancer	15	2	49
Rectosigmoid	39	3	22
Prostate cancer	2	4	21
Renal cell carcinoma	1	5	7
Adrenal gland	1	6	4
Gall bladder cancer	1	>6	3
NSCLC	5	PTV diameter (mean, SD)	3.2 ± 1.9 cm
Ovarian cancer	3	PTV volume (mean, SD)	23.3 ± 58.1 ccm
Other metastatic sites	45	PTV dose coverage (mean, SD)	
Pulmonary	21	V100	23 ± 58 ccm
Bone	11	V98	23 ± 57 ccm
Lymph nodes	18	V95	22 ± 55 ccm
Others	6	V90	21 ± 52 ccm
Prior local treatment liver	56	D100	19 ± 5 Gy
Multiple	14	D98	23 ± 6 Gy
Surgery	37	D95	25 ± 6 Gy
Other LAT	33	D90	28 ± 7 Gy

**Table 2:** Literature review of interstitial-brachytherapy of liver metastases.

Study (Year)	Entity	Patients	Lesion Count	Lesion Size (cm)	Applied Dose (D100)	LTCR at FU (Months)	OS Median
Ricke (2010) [17]	CRC	73	200	3.6 (1–13.5)	3 dose groups 12.8–18.8 Gy	74.9% at 34 m	23.4 m (26 m with CTX)
Wieners (2009) [22]	CRC	33	138	4.6 (1–12)	3 dose groups 14.2–21.2 Gy	87% at 6 m 76% at 12 m 69% at 24 m	n/a
Collettini (2014) [23]	CRC	80	179	2.85 (0.8–10.7)	19.1 Gy (15–20 Gy)	88.3% at 12 m81.2% at 24 m68% at 36 m	18 m
Wieners (2011) [24]	Breast cancer	41	115	4.4 (1.0–11)	18.5 Gy (12–25 Gy)	97% at 6 m 93.5% at 18 m	n/a
Geisel (2013) [25]	Renal cell carcinoma	10	16	3.8 (1–8.2)	n/a	93.8% at avg. FU 21.6 m ±13.7 m)	n/a
Geisel (2012) [26]	Gastro-esophageal cancer	8	12	4.6 (1.4–6.8)	21 Gy (5–29 Gy)	100% at 8.4 m (±6.8 m FU)	n/a
Collettini (2012) [27]	Breast cancer	37	80	2.6 (0.8–7.4)	18.6 Gy (±2.27 Gy)	94.6% at 12 m	18 m (3–39 m)
Wieners (2015) [28]	Pancreatic cancer	41	49	2 (1.0–7.3)	18.1 Gy	91% at 12 m	8.6 m (1.5–55.3 m)
Schippers (2016) [29]	NET	27	52	2.1 (0.7–11.0)	15–20 Gy (target)	92% at 1 y 83% at 3 y	36 m (4.2–106.1 m)
Omari (2018) [30]	GIST	10	40	2.4 (0.6–11.2)	15 Gy (6.7–21.96 Gy)	97.5% at 25 m median FU	37.3 m (11.4–89.7 m)
Heinze (2018)	Anal cancer	7	38	1.2 (0.4–6.2)	16.2 (12.0–32.6 Gy)	97.4% at 15 m	25.2 m (6.5–51 m)
Kieszko (2018) [31]	Mixed histologies	61	73	<8	13 Gy (7–20 Gy)	88.7% at 6 m 70.7% at 12 m	96.7% at 6 m 79.6% at 12 m
Omari (2019) [32]	Gastric cancer	12	36	2 (1–10.2)	19.9 Gy (5.4–22.5 Gy)	89% at 8.3 m median FU	11.4 m (4.3–47 m)
Omari (2019) [33]	Renal cell carcinoma	14	54	1.8 (0.5–13.9)	16.1 Gy	92.6% avg. FU 10.2 (range 2.4–73.6 m)	51.2 m (1.0–27.8 m)
Drewes (2019) [34]	Pancreatic cancer	16	45	2.2 (1.0–11.2)	21 Gy (5–29.1 Gy)	87% at 3.3 m median FU	8.9 m (3.1–29.3 m)
Heinze (2020) [35]	Mixed histologies	59	194	A:1.6 (0.4–3.8) B:6 (4–13.9)	17.1 Gy (5–29.1 Gy)	90.2% at 6 m median FU	n/a

## Data Availability

The data presented in this study are available on request from the corresponding author.
